# Chemoprevention with the metabolism modifying drugs dichloroacetate and metformin in Trp53+/- mice

**DOI:** 10.1186/1897-4287-10-S2-A62

**Published:** 2012-04-12

**Authors:** AC Blackburn, M Rooke, Y Li, JE Dahlstrom, PG Board

**Affiliations:** 1 Molecular Genetics Group, John Curtin School of Medical Research, Australian National University, Canberra ACT 0200, Australia; 2 Department of Anatomical Pathology, Canberra Hospital, Woden ACT 2606, Australia

## 

While genetic testing for familial cancer susceptibility factors has excelled in recent years, the prevention options for those carrying high risk alleles have not. Altered bioenergetics has now been acknowledged as an emerging hallmark of cancer, and is believed to be a critical characteristic acquired during tumorigenesis. Several very safe drugs are available that can modify bioenergetics. Dichloroacetate (DCA) inactivates pyruvate dehydrogenase kinase, resulting in activation of pyruvate dehydrogenase, reduced lactic acid production and increased mitochondrial activity. Metformin, a type 2 diabetes treatment which activates AMPK, thereby inhibiting mTOR, has unambiguously been demonstrated to reduce the risk of many cancer types in diabetics where insulin treatments do not. We have tested these drugs as chemopreventive agents against the mammary tumours that occur in the spontaneous BALB/c-*Trp53*+*/-* mouse tumour model.

In vitro studies on breast cancer cell lines indicate that DCA (1-5 mM), metformin (30-300 uM) or the combination of the two can significantly inhibit breast cancer cell growth over 4 days of treatment. These results support the possibility that DCA and metformin could prevent or delay breast cancer formation in BALB/c-*Trp53*+*/-* mice. To examine this, four groups of female BALB/c-*Trp53*+*/-* mice were given distilled water (n=75), DCA (1.5 g/L in drinking water, ~180 mg/kg/day, n=53), metformin (0.25 g/L in drinking water, ~30 mg/kg/day, n=61) or DCA + metformin (n=51) from 8 weeks of age, and monitored for tumour development over 78 weeks. The overall tumour-free survival curves were not significantly different (Kaplan-Meier analysis) between the treatment groups, suggesting that metformin does not reduce cancer risk in non-diabetics. However, the occurrence of mammary tumours in the four groups was altered. DCA reduced the number and increased the latency of mammary tumours (20.8% of DCA treated mice with mean latency of 63.8 weeks compared to 28.0% of untreated mice with mean latency of 55.0 weeks), whereas metformin had no effect (26.2% of mice, average latency 54.7 weeks). Examining the mammary tumour-free survival curves, DCA appeared to eliminate the early onset mammary tumours (latency <52 weeks, p=0.02), while not affecting the occurrence of longer latency tumours. We are currently examining these tumours to identify characteristics that may explain this difference in sensitivity to DCA prevention. Contrasting with the in vitro cancer cell line result, the two drug combination had worse outcomes for tumour development, with 35.3% of mice developing mammary tumours with a decreased latency of 48.8 weeks (p<0.02 compared to DCA alone). Preliminary western blotting results in MDA-MB-468 breast cancer cells found that DCA could block the activation of AMPK by metformin, indicating the potential for drug interactions. However, the outcomes of these drug interactions clearly differs in the fully transformed cancer cell (growth inhibition) compared to the preneoplastic cell (survival / growth advantage) and requires further investigation.

